# The role of laparoscopy in closed abdominal injury

**DOI:** 10.1016/j.heliyon.2023.e20705

**Published:** 2023-10-05

**Authors:** Jing-nan Fu, Li Zhou, Tao Ma

**Affiliations:** a Department of Minimally Invasive Surgery, Characteristics Medical Center of Chinese People Armed Police Force, Tianjin, China; b Department of General Surgery, Tianjin Medical University General Hospital, Tianjin, China; c Department of nephrology, Characteristics Medical Center of Chinese People Armed Police Force, Tianjin, China

**Keywords:** Laparoscopic technique, Abdominal closed injury, Diagnosis, Treatment

## Abstract

**Objective:**

To investigate the effect of laparoscopy on the diagnosis and treatment of closed abdominal injury.

**Methods:**

A total of 26 patients with closed abdominal injury admitted to our hospital from January 2016 to January 2019 were searched. All patients were treated by laparoscopy. All patient reports were made with the informed consent of the patients.

**Results:**

All patients were diagnosed clearly during operation. Among them, there were 3 cases of gastric perforation, 2 cases of liver rupture, 13 cases of spleen rupture, 3 cases of small intestine rupture, 1 case of liver round ligament laceration, 2 cases of small mesenteric vascular laceration, 1 case of colon liver mesenteric laceration, and 1 case of retroperitoneal hematoma. Of the 26 patients in this group, 23 (88.5%) completed laparoscopically or laparoscopically assisted; 5 cases (19.3%) only performed laparoscopic exploration without special treatment, and 3 cases (11.5%) switched to laparotomy. The blood loss was 50–2000 ml (mean 500 ml), and the operative time was 60–180min (mean 128min). The length of hospital stay was 3–21 d (mean 9 d). There were no complications or deaths related to laparoscopy in the whole group. Conclusion Laparoscopic technique has the advantages of less trauma, high diagnosis rate and fast recovery. It can also be used for surgical treatment in the diagnosis and treatment of closed abdominal injury, so as to achieve the purpose of comprehensive diagnosis and treatment. The limitations of laparoscopy should also be noted.

## Highlights

• A lot of studies evaluating laparoscopy for trauma were published before, but the role of laparoscopy in the evaluation of abdominal trauma is still unclear. There are no prospective randomised studies comparing laparoscopic surgery and an open procedure in patients with abdominal trauma. The majority of publications on this subject are retrospective single-centre studies. Laparoscopy has not only gained popularity in elective surgery during recent years but its use in hemodynamically stable trauma patients has also increased. In this manuscript, we focus on the role of laparoscopic techniques in the treatment of abdominal injuries.
• All authors declare that they have no known competing financial interests or personal relationships that could have appeared to influence the work reported in this paper.

## 1 Introduction

In abdominal trauma, closed abdominal injury is the most common type in clinical abdominal trauma, the condition changes quickly, and it is easy to produce misdiagnosis phenomenon. In the emergency department of general surgery, the incidence of closed abdominal injury is relatively high, and early diagnosis is difficult. Many patients have atypical early symptoms without obvious signs of peritonitis and hemorrhagic shock, which often make surgeons entangled in continued observation and immediate operation [1]. In the past, the treatment was strictly in accordance with surgical indications, conservative treatment and observation were performed when there was no signs of peritonitis or hemorrhagic shock, and the patient had no obvious symptoms of discomfort in the early stage. When surgical indications appeared, the operation was conducted in time, but there was a risk of delay and aggravation of the disease during the waiting period. Most of the previous surgical exploration used traditional open surgery, often the incision distance is far from the injured site, it is difficult to see the injury location, need to extend the knife edge and expand the incision, undoubtedly increase the risk of incision infection and postoperative recovery time of patients [2,3]. Laparoscopy can not only diagnose patients with abdominal injury with unclear early diagnosis, but also carry out hemostasis, suture, repair, anastomosis, resection and other means, so as to achieve integrated diagnosis and treatment [4]. For patients who need to switch to open surgery, laparoscopy can also select the best surgical incision for them [5]. We searched 26 patients with closed abdominal injury admitted to our hospital from January 2016 to January 2019, all of whom were diagnosed and treated by laparoscopy, and the therapeutic effect was good.

## 2 Methods

The selected 26 patients came to the hospital with relatively stable condition, no hemorrhagic shock, coma and other manifestations, and no contraindications to laparoscopic surgery. Before surgery, we need to assess the patient's hemodynamic status, including the presence of hypoperfusion and the presence of fluid responsiveness. The perfusion was evaluated by capillary refilling, shock window, blood gas lactate, central venous oxygen saturation and venous-arterial CO_2_ gradient. CVP, ultrasound and transpulmonary thermodilution were used to determine the volume status of patients, and fluid challenge test was used to determine whether the patients had fluid responsiveness. Laparoscopic operation: conventional CO_2_ pneumoperitoneum was established above or below the umbilical cord, and the pressure was controlled at 12 mmHg. Laparoscopic abdominal exploration was performed to understand the damage of abdominal organs, abdominal bleeding, injury site and injury degree. The surgical method is determined according to the injury condition. Abdominal blood and blood clots are cleaned by suction device. For abdominal bleeding, vascular clamp, hemostatic gauze compression, ultrasonic knife and electric coagulation can be used. For abdominal organ injury, repair or organ excision is used. When laparoscopic management is difficult, open surgery should be transferred in time. When combined with other injuries, relevant departments should be consulted for assistance in treatment. In the operation of these patients, the surgical duration varied from 30 min to 1 h for five patients, from 1 h to 2 h for fifteen patients, and from 2 h to 3 h for six patients.

In the above patients with closed abdominal injuries, the patients with gastric perforation decided to undergo laparoscopic repair of gastric perforation or gastrectomy according to the size, degree and location of the perforation. Hepatic rupture was performed according to the criteria of the Organ Injury Scoring Committee of the American Society for Traumatic Surgery (AAST) [6]. Splenic rupture should be treated according to the grading standards established by the splenic Surgery Group of the Chinese Surgical Society in 2000 [7]. Splenic rupture bleeding is large, fierce and fast, and immediately transferred to open splenectomy; The spleen was slightly lacerated with less bleeding. Hemostasis could be performed by means of electric coagulation, hemostatic gauze compression and biological glue spraying after blood accumulation was sucked out by suction device. Laparoscopic splenectomy is feasible for grade Ⅱsplenectomy, if the amount of bleeding is not large. If the patient only sees a small amount of abdominal hemoperitoneum and mild contusion of spleen, only abdominal drainage tube is placed without special treatment. Intestinal rupture according to the perforation site laparoscopic intestinal rupture repair or intestinal resection; For hepatic round ligament laceration, the specific operation method is determined according to the size of the laceration. If the laceration is small, vascular clamp clamp hemostasis and ultrasonic knife electrocoagulation hemostasis are performed. If laceration of mesocolon and mesocolon vessels is limited, laparoscopic vascular clamp clamp should be performed to stop bleeding. Retroperitoneal hematoma patients according to the size of the hematoma to determine the specific operation.

### 2.1 Results

Among the 26 patients with closed abdominal injury, 18 were males and 8 were females, ranging in age from 18 to 68 years, with an average age of 35.5 years. There were 16 cases of car accident trauma, 5 cases of blunt force trauma, 4 cases of height fall injury and 1 case of crush injury. The time from injury to hospital admission was 1–12 h (mean 5 h). Preoperative CT and color ultrasound examination showed that there were 21 cases of abdominal effusion, 13 cases of spleen contusion, 2 cases of liver contusion, and 1 case of retroperitoneal hematoma. There were 5 cases with no abdominal effusion, 6 cases with abdominal effusion <200 ml, 7 cases with 200 ml–500ml, and 8 cases with 500 ml–1000ml.

Of the 26 patients in this group, 23 (88.5%) completed laparoscopically or laparoscopically assisted; 5 cases (19.3%) only performed laparoscopic exploration without special treatment; Laparoscopic mesenteric ligation hemostasis was performed in 2 cases (7.7%).1. Laparoscopic mesangial ligation of colon and liver was performed to stop bleeding. Three cases (11.5%) underwent laparoscopic repair of gastric perforation. Laparoscopic liver rupture repair was performed in 2 cases (7.7%). 4 cases (15.4%) underwent laparoscopic splenectomy; Local hemostasis of splenic laceration was performed in 2 cases (7.7%). One case (3.8%) underwent laparoscopic hepatic round ligament clamp hemostasis. Three cases (11.5%) underwent laparoscopic repair of intestinal rupture; Three cases (11.5%) were converted to laparotomy. Among the 23 patients who underwent laparoscopic or laparoscopic-assisted splenectomy, only 1 patient had bleeding incision infection, and among the 3 patients who switched to open splenectomy, only 1 patient had bleeding incision infection. (Table 1). The blood loss was 50–2000 ml (mean 500 ml), and the operative time was 60–180min (mean 128min). The length of hospital stay was 3-21 d (mean 9 d). There were no bleeding laparoscopic complications or deaths in all patients.

**Table 1:** Surgical methods and effects of patients (n = 26).

modus operandi	number of cases	constituent ratio（%）	result
Done laparoscopically or laparoscopically assisted	23	88.5	recovery
Simple laparoscopic exploration	5	19.3	recovery
Laparoscopic mesenteric ligation hemostasis	2	7.7	recovery
Laparoscopic colohepatic mesangial ligation hemostasis	1	3.8	recovery
Laparoscopic repair of gastric perforation	3	11.5	recovery
Laparoscopic liver rupture repair	2	7.7	recovery
Laparoscopic splenectomy	4	15.4	recovery
Laparoscopic local hemostasis of splenic laceration	2	7.7	recovery
Laparoscopic hepatic round ligament clamp hemostasis	1	3.8	recovery
Laparoscopic repair of intestinal rupture	3	11.5	One case of incision infection
Complete conversion to open surgery	3	11.5	recovery
splenectomy	3	11.5	One case of incision infection

There was 1 case of incision infection during laparoscopic intestinal rupture repair, and Clavien-Dindo classification was grade I. During splenectomy, one patient also had incision infection, which was also classified as grade I according to the Clavien-Dindo classification system [8].

## 3 Discussion

Closed abdominal injury refers to the patient's abdominal injury, but it does not penetrate the abdominal wall or communicate with the outside world. It is a common surgical disease in clinic. In clinic, closed abdominal injury is mainly caused by blunt injuries such as falling from high altitude, batting, abdominal impact and squeezing, and the number of patients is about 78.9%–95.6% [9,10]. Closed abdominal injury usually affects the injury of liver, colon and other organs [11]. The prognosis depends on the presence of hemorrhagic shock, organ injury and abdominal great blood vessel injury. Most closed abdominal injuries are combined with multiple injuries, such as abdominal trauma combined with brain trauma, abdominal trauma combined with rib fracture, etc., with complicated clinical treatment, most of which require multidisciplinary consultation and treatment [12,13]. If it is not possible to determine whether there is organ damage in patients with closed abdominal injury, exploratory laparotomy can be performed to check, but it will increase the mortality of patients, make patients produce postoperative complications, and also affect the necessary surgical items of patients, seriously endanger life and health. The main causes of early death were hemorrhagic shock and septic shock, and the patients often died of massive abdominal hemorrhage without time for operation.

In clinical diagnosis of closed abdominal injury, according to the patient's disease history, the patient's symptoms of abdominal injury, hemorrhagic shock, peritonitis, abdominal lavage, as well as the relevant diagnostic basis of medical photography, B-ultrasound, CT, etc., it is easier for doctors to diagnose the patient's condition. But there are still many factors that lead to inaccurate diagnosis and pain for patients. Even with a clear diagnosis, it is difficult to determine the severity of the injury and how to manage the wound. Laparoscopy can directly view the lesions through small wounds, which is convenient for doctors to observe whether there is damage to patients' internal abdominal organs, understand the severity of patients' injuries, and judge how to treat patients. The use of laparoscopy brings more convenience to doctors.

As an important part of minimally invasive techniques, laparoscopy plays an increasingly prominent role in closed abdominal injuries [14]. Laparoscope can be used for direct vision and enlargement of abdominal organs, and can be used for suture, clamp, hemostasis, excision and other treatments under laparoscope. Spraying hemostatic glue, hemostatic gauze compression and suture hemostasis can all be implemented under laparoscopy. Compared with traditional open surgery, laparoscopic surgery has the advantages of less trauma, less postoperative pain and faster recovery. It is suitable for patients with low organ function and poor wound repair and healing ability. Age is not a decisive factor in laparoscopic surgery [15]. Elderly patients with good cardiopulmonary function and no serious organic changes can be given priority to laparoscopic surgery. Preoperative attention should be paid to the patient's cardiopulmonary function, although laparoscopic surgery has less trauma and less interference to patients, but anesthesia, surgery and other shocks can still have a serious impact on the heart, lung, kidney and other important organs [16]. The main manifestations of hemodynamic instability were insufficient blood supply to the heart, insufficient blood supply to the brain, and abnormal pumping of the heart [17]. The establishment of pneumoperitoneum in patients with organ injury and hemodynamic instability may further aggravate the hypoxic-ischemic injury of heart, lung, liver, kidney and other organs [18]. Therefore, most hemodynamically unstable patients are not candidates for laparoscopic surgery.

Laparoscopic techniques can be used for a variety of abdominal surgical injuries. For patients with liver and spleen rupture, laparoscopy can also be used to perform corresponding liver and spleen resection. If the patient's wound is deep and the amount of bleeding is large, laparotomy can be performed on the patient immediately [19]. Patients with gastric perforation can also undergo repair and resection of gastric perforation under laparoscopy. The specific surgical method is determined according to the patient's condition and resection site. Patients with intestinal perforation should be judged according to the size of their intestinal perforation and the extent of their intestinal damage. Patients with mild cases can be repaired and excised by laparoscopy [20]. Laparoscopy can also perform corresponding ultrasonic knife hemostasis, hemostatic clamp hemostasis and suture hemostasis for patients suffering from laceration of mesentery and mesentery [21]. If it is found in laparoscopic exploration that the degree of injury is relatively serious and the amount of blood loss is large and difficult to manage, the patient can be operated on immediately. Through laparoscopic exploration, doctors can better select an appropriate incision site and a reasonable surgical plan for open surgery. All the patients in this group have used the above methods, and the effect is satisfactory.

Although laparoscopy has obvious advantages in the diagnosis and treatment of closed abdominal injuries, it also has many limitations [22]. Such as flatulence, abdominal adhesion and other reasons will affect the laparoscopic visual field. For deep-seated organ injuries, such as pancreas and duodenum injuries, laparoscopic exposure is difficult and visual field blindness exists, so it is necessary to switch to laparotomy in time. Secondly, when the amount of abdominal bleeding is large and the bleeding is dangerous and the laparoscopy is difficult to deal with, it is also necessary to switch to laparotomy immediately to ensure safety.

## Funding sources

A portion of this work was funded by the Tianjin Key Medical Discipline (Specialty) Construction Project.

## Data availability statement

Data included in article/supplementary material/referenced in article.

## Additional information

No additional information is available for this paper.

## CRediT authorship contribution statement

**Jing-nan Fu:** Software, Supervision, Visualization. **Li Zhou:** Data curation, Methodology, Project administration. **Tao Ma:** Conceptualization, Investigation.

## Declaration of competing interest

The authors declare that they have no known competing financial interests or personal relationships that could have appeared to influence the work reported in this paper.
